# Cytomolecular and In Vitro Toxicity Studies on Bisphenol A Effect on Ovine Granulosa Cells

**DOI:** 10.1155/vmi/5498627

**Published:** 2026-02-13

**Authors:** Poonam Kumari Singh, Bogapathi Sampath Kumar, Sumanta Nandi, Paluru Subramniyam Parameswara Gupta, Sukanta Mondal

**Affiliations:** ^1^ Jain University, Bangalore, India, jainuniversity.ac.in; ^2^ ICAR-National Institute of Animal Nutrition and Physiology, Adugodi, Bangalore, India

**Keywords:** apoptosis, bisphenol A, GCs, steroidogenesis, viability

## Abstract

Bisphenol A (BPA), an organic environment chemical, has extensive presence in consumer goods and food/feed items. The present study aimed to explore how BPA influenced the viability, proliferation, apoptosis, and steroidogenic activity of ovine ovarian granulosa cells (GCs). GCs were isolated from ovine ovaries obtained from a local abattoir and were cultured for 2, 3, and 6 days in the presence of varying BPA concentrations (0, 1, 10, 25, 50, and 100 μm). The proliferation and cytotoxicity of the GCs were evaluated using kit assays, and the 72‐h culture’s spent media were pooled to measure the hormone concentrations. qPCR was performed for the study of gene expression–related apoptosis and steroidogenesis. For further confirmation of viability and apoptosis, the trypan blue exclusion test and Hoechst staining were performed. The findings revealed that the metabolic activity was significantly reduced at 50 μm, while the cell viability dropped notably (*p* < 0.05) at concentrations of 10 μm and above. Hormonal analysis indicated a biphasic response: estrogen and progesterone levels were significantly elevated at lower BPA concentrations (1 μm) but reduced from 25 μm onwards. Cytotoxicity assessments showed marked changes in LDH and GST activity at 50 μm and increased MDA and ROS levels at 25 and 10 μm. However, total antioxidant activity (CUPRAC) remained unchanged compared to control samples. Gene expression analysis revealed a significant upregulation of *ESR1*, *ESR2*, *PGR*, and *FSHR* at 1 μm; *BAX* and *CASP3* at 25 μm; and *17βHSD* and *BCL2* at 50 μm. Conversely, a significant downregulation was observed for *3βHSD1* at 1 μm, *CYP19A1* at 50 μm, and *StAR* at 100 μm, with no notable changes in *CYP11A1* and *CYP17A1*. Overall, the study demonstrated that BPA adversely affects GCs by disrupting their growth, steroidogenic function, and gene expression, exhibiting estrogenic effects at lower doses and suppressing hormone secretion at higher concentrations.

## 1. Introduction

Environmental chemicals can disrupt granulosa cell (GC) steroidogenesis, potentially impacting the oocyte quality and female reproductive success [1, 2]. This highlights the sensitivity of GC function to external influences and underscores the importance of maintaining a supportive environment for optimal reproductive health.

Among the environmental chemicals, bisphenol A (BPA), a significant endocrine disruptor with a structure similar to synthetic estrogen, is extensively used as a plasticizer in polycarbonate monomers, epoxy resins, and thermal papers due to its heat resistance and flexibility [3, 4]. Humans and other animals are typically exposed to BPA because it depolymerizes during manufacturing processes and leaches from the materials [5]. BPA has been detected in bovine milk [6], bovine urine [7], women amniotic fluid, and fetal serum [8]. Assessing the effects of environmental chemicals in a domestic animal species relevant to human female reproduction is crucial [9]. Sheep have also been recognized as a suitable model for toxicology studies, including those on BPA [10, 11].

BPA has emerged as a significant environmental contaminant that disrupts cellular functions and reproductive performance in mammals [12]. BPA’s detrimental effects on bovine fertility have garnered increasing attention, highlighting the need for a comprehensive understanding of its mechanisms of action [13, 14]. Ovarian GCs are essential for oocyte development and are particularly sensitive to endocrine disruptors such as BPA and its analog, bisphenol S (BPS) [15]. BPA was reported to induce apoptosis in bovine GCs through intrinsic pathways, marked by an increase in apoptosis‐related transcripts and proteins, a reduction in live cell counts, and an elevated BAX/BCL2 ratio [16]. Similarly, investigation on the impact of BPA, BPS, and the membrane estrogen receptor (GPER) on ovine GC steroidogenesis revealed that BPA exhibited a higher affinity for GPER compared to estrogen receptors (ERα and ERβ) [17]. Additionally, research conducted to assess the effects of BPS on the ewe oocyte quality during in vitro maturation found that low doses of BPS significantly reduced cleavage and blastocyst rates, decreased oocyte maturation, and lowered cumulus cell progesterone secretion [18]. Moreover, BPA’s impact on GC development and oocyte meiosis through in vitro culture of mouse preantral follicles [19] was investigated, and it was found that BPA enhanced proliferating cell nuclear antigen (PCNA) and estradiol receptor α (ERα) expression and increased estrogen and progesterone secretion, which simultaneously impaired cumulus cell expansion and disrupted gap junction communication GCs. These findings collectively highlight the critical need to explore the impacts of BPA on reproductive health and underscore the importance of regulatory measures to mitigate their effects.

Additionally, recent ovine‐focused studies have shown that both BPA and its common substitute BPS adversely affected GC function in sheep. BPA and BPS impaired steroidogenesis in ovine GCs, reducing progesterone secretion and altering the expression of key steroidogenic genes [11]. Both bisphenols disrupt steroidogenesis via partially distinct molecular pathways involving oxidative stress and differential engagement of ERα/ERβ and GPER [17]. Furthermore, BPS decreased cumulus expansion and progesterone secretion during in vitro oocyte maturation in ewes, indicating negative effects on oocyte competence [18]. Toxicokinetic study further supported the reproductive relevance of these exposures in sheep by showing placental transfer and fetal tissue accumulation [10].

Thus, the present study aimed to investigate the effects of different concentrations of BPA on the morphological functions (viability, cell number increment, and monolayer formation) and metabolic activities of GCs, as well as their impact on reactive oxygen species (ROS) production, lipid peroxidation, cytotoxicity assessed via lactate dehydrogenase (LDH), and steroid production (estrogen and progesterone) and the expression of key genes associated with apoptosis and steroidogenesis in ovine GCs.

## 2. Materials and Methods

### 2.1. Chemicals and Reagents

BPA, DMSO, trypan blue, Hoechst 33342, and primers were purchased from Sigma Chemical Co., Ltd (St. Louis, MO, USA) unless otherwise treated. OxiSelect Hydrogen Peroxide/Peroxidase Assay Kit (Cat no. STA‐844) was from Cell Biolabs Inc. (San Diego, California). Tris–HCl, EDTA, Triton X‐100, PBS, molecular grade water, 3‐(4,5‐dimethylthiazol‐2‐yl‐2,5‐diphenyl tetrazolium bromide (MTT), LDH, malondialdehyde (MDA) assay kits, glutathione‐S‐transferase (GST), cupric reducing antioxidant capacity (CUPRAC) kits were bought from HiMedia (Mumbai, India). Calbiotech, Mumbai, India, provided 17β‐estradiol and progesterone ELISA kits. RNA Sure Mini kit (Genetix, India), SYBR green, and a cDNA synthesis kit were from Bio‐Rad, USA. The plasticware was purchased from Tarsons, Kolkata, India.

### 2.2. BPA Treatment

A solution of BPA (3.38 mg) was prepared by diluting it with 5 mL of DMSO to achieve a concentration of 1 mm. This solution was stored at −20°C until needed. During all exposure experiments, the DMSO content was consistently maintained at 0.1% and there was no negative impact on the cells, and therefore, the same concentration was maintained in the control condition [20]. GCs were exposed to BPA at varying concentrations of 0, 1, 10, 25, 50, and 100 μm. The BPA dosage was selected based on previous research studies on the effects of environmental chemicals on GC functions [11, 21].

### 2.3. GC Collection, Processing, Culture, and Evaluation of Viability, Cell Number Increment, and Monolayer Formation

The methods for GC isolation, culture, assessing the metabolic activity (after 3 days of culture), viability (after 3 days of culture), cell proliferation (after 2 days of culture), and monolayer formation (after 5 days of culture) were adapted from previous studies conducted in our laboratory [22, 23]. In brief, ovine ovaries (*n* = 265) were collected from a slaughterhouse in Bangalore, India, and immediately placed in sterile 0.9% saline containing 50 μg/mL gentamicin. The ovaries were rinsed three times with normal saline and cleansed with 75% ethanol for 3–5 s. Ovarian follicles were then punctured with a 22 G sterile needle attached to a syringe, and the follicular fluid was aspirated from all surface follicles into aspiration media consisting of TCM 199, gentamicin (50 μg/mL), and heparin (10 μg/μL). The aspirated follicular fluid, containing a mixture of GCs and oocytes, was subsequently filtered through 40‐μm cell strainers to separate the components. The GCs were centrifuged at 400 g for 5 min at 4°C, resuspended in 1% PBS, and washed three times in TCM 199 supplemented with gentamicin (50 μg/mL) and 0.3% BSA. They were then centrifuged at 200*g* for 10 min and finally resuspended in 1 mL of TCM 199 with 0.3% BSA. The viability and culture of the GCs were assessed as previously described [22]. The cells were cultured for 72 h at 37°C in a CO_2_ incubator with 5% CO_2_ and 95% humidity. The growth of the GCs was examined under a microscope every other day, and fresh media was added accordingly. The monolayer formation in GCs was evaluated over a 5‐day period and scored based on the following criteria [22]: Score 3, monolayer formation began by Day 2 and covered most of the droplet by Day 5; Score 2, monolayer formation began by Day 3 and covered more than 50% of the droplet by Day 5; Score 1, monolayer formation began by Day 4 and covered less than 50% of the droplet by Day 5; and Score 0, no monolayer formation by Day 4. The experiment was replicated 12 times, with each replicate using three or four droplets per treatment. In a separate experiment, GCs (1.0 × 10^5^ cells per droplet) were cultured for 2 days, harvested, and the increase in cell number was monitored by counting cells with a hemocytometer. Cell viability after culture was determined using the trypan blue exclusion test. This experiment was also replicated 12 times, with each replicate using one or two droplets per treatment.

### 2.4. Preparation of GC Lysis Supernatant

Following a 3‐day culture period, the isolated GCs from all experimental conditions were individually homogenized using a sonicator. Each sample underwent three 15 s sonication cycles at 50 W, with 5‐s intervals, in 400 μL of 1X lysis buffer (pH 8) containing 10 mm Tris–HCl, 20 mm EDTA, and 0.25% v/v Triton X‐100. Subsequently, the cell lysates were centrifuged at 10000 × g for 20 min at 4°C to obtain the supernatants for biochemical analysis.

### 2.5. Hoechst Staining

To assess and confirm cellular apoptosis, Hoechst staining was conducted as described earlier [24]. BPA‐treated and untreated GCs were cultured for 3 days. Control GCs were fixed in 4% paraformaldehyde, stained with Hoechst 33342 (5 μg/mL) for 20 min at room temperature, and subsequently washed. The stained cells were mounted on glass slides and examined by fluorescence microscopy. This procedure was performed in triplicate and repeated three times to ensure consistency. The morphology of the nucleus was examined under fluorescence microscopy (Nikon 80i Eclipse, Japan) to identify cells undergoing apoptosis.

### 2.6. GC Metabolic Activity, Cytotoxicity, Oxidative Stress, and Lipid Peroxidation

The viability percentage (trypan blue 0.4%), metabolic activity, cytotoxicity, and oxidative stress of BPA‐treated and control GCs were evaluated using the MTT, LDH, ROS, and MDA assays using commercial kits and according to the manufacturer’s protocol.

### 2.7. MTT Assay

GCs were harvested on the third day and washed with PBS. The MTT solution (provided in the kit) was added to each well cell and incubated for 4 h. After incubation, a solubilization solution (provided in the kit) was added to dissolve formazon crystals, and finally, absorbance was measured at 570 nm using an ELISA microplate reader. The absorbance intensity at 570 nm directly reflects the cell metabolic activity (darker the purple color, more metabolically cell). All experiments were replicated six times in three replicates for accuracy.

### 2.8. LDH Assay

Three‐day‐cultured GCs were incubated for 4–5 h in a CO_2_ incubator, and lysis solution was added to control wells for maximum LDH control, DMSO to untreated control wells, BPA to experimental wells, and PBS to background control wells; the microplate was then incubated for 8 h, and after incubation, the LDH reagent was added before measuring absorbance at 580 nm. The percentage cytotoxicity was calculated as per manufacturer’s protocol. The experiments were replicated six times in three replicates for accuracy.

### 2.9. ROS Determination by Hydrogen Peroxide (H_2_O_2_) Kit

GCs were harvested after 3 days of culture and washed twice with PBS and sonicated to lyse cells, and the resulting homogenate was collected and analyzed for ROS content in the samples. Briefly, this assay involves the reaction of a probe with H_2_O_2_ in the presence of hydrogen peroxidase that gives a bright pink color. The absorbance can be measured at 540 nm and values in the absorbance correlating to H_2_O_2_ levels. The H_2_O_2_ concentration (μm) in unknown samples was determined by standard curves initially prepared using H_2_O_2_ standards. The H_2_O_2_ stock (given in the kit) was diluted to 1:1000 in 1X assay buffer. Subsequently, controls and unknowns were assessed in triplicate, each containing 50 μL of H_2_O_2_ working solution and 50 μL of cell lysate. After an incubation period of 30 min at 25°C, the absorbance was measured at 540 nm using a microplate reader, and the H_2_O_2_ concentration (μm) in the unknown samples was determined by comparing the absorbance values to the standard curve.

### 2.10. MDA Assay

GCs were harvested after 3 days of culture, washed, and sonicated, and the resulting cell lysate was used for further analysis. Briefly, glass tubes were labeled standards, blank control, and sample. Reagents and color development solution (provided in the kit) were added to each glass tube. MDA standards ranging from 2 to 20 mm, blank control (distilled water), and samples were added to the corresponding tubes. Glass tubes were covered with foil and incubated in a boiling water bath (95°C) for 70 min. Glass tubes were cooled in an ice bath and then centrifuged. After cooling, samples were transferred to into a 96‐well plate and measured at 532 nm using a microplate reader. The MDA concentration was determined in μm based on the absorbance.

### 2.11. 17β‐Estradiol and Progesterone Estimation by ELISA

The 17β‐estradiol and progesterone in GC spent media (3 days of culture) were examined by the ELISA method. The method and protocol were followed according to the manufacturer’s instructions. The estradiol ELISA, estrogen standards, controls, and samples (spent medium) were incubated with estrogen enzyme conjugate (given in the kit), and it competed with endogenous estrogen for binding sites on the specific estrogen antibody coated in the well already. After incubation for 45 min, all wells were washed and treated with the TMB reagent and incubated for 60 min. After the incubation period, the stop solution was added. Absorbance was measured at 450 nm using a microplate reader. The results were calculated from an estrogen standard curve according to instructions given in the kit, and the concentration of estradiol was determined in pg/mL from the standard curve. All the measurements were taken in triplicate. The intra‐ and interassay coefficients of variation were below 5% for all the analyses. Similarly, progesterone ELISA and streptavidin‐antibody‐coated wells were used for the progesterone content in the samples.

Progesterone enzyme (HRP) conjugate and the antiprogesterone‐biotin reagent were added to the wells and incubated for 45 min. After incubation, unbound progesterone was washed off from the well and the TMB substrate was added, which produced a color development, which is inversely proportional to the progesterone concentration in the sample. The standard curve was prepared to correlate the color intensity to the progesterone concentration in the sample. The results were calculated from an progesterone standard curve. All the measurements were taken in triplicate, and the progesterone concentration was measured in ng/mL. Intra‐ and interassay coefficients of variation were below 5%.

### 2.12. Gene Expression Studies

Gene expression studies related to hormone receptor genes (*ESR1, ESR2, PGR*, and, *FSHR*), steroidogenic genes (*StAR, 3βHSD1, 17βHSD1, CYP11A1, CYP17A1,* and *CYP19A1*), and apoptosis‐related genes (*BAX, BCL,* and *CASP3*) were performed for evaluating the effect of BPA on the GCs.

### 2.13. RNA Isolation

RNA isolation was performed from the GCs that were cultured for 72 h. GCs were washed twice with PBS and centrifuged at 800 × *g* for 15 min, and the supernatant was decanted and the cell pellets were kept for the RNA isolation. Total RNA was extracted from the RNA Sure Mini kit (Genetix) from the cells according to the manufacturer’s protocol. The RNA concentration was calculated using a nanodrop to measure the purity of the samples, and protein impurities were calculated using absorbance values at 260/280 and 230/280 nm.

### 2.14. Real‐Time Reverse Transcription Polymerase Chain Reaction (RT‐PCR)

Total RNA from the GCs (100 ng/sample) were converted into cDNA using the Bio‐Rad iScript^TM^ cDNA Synthesis kit according to the manufacturer’s protocol. RT‐PCR was performed using iTaq Universal SYBR Green Supermix (Bio‐Rad). Master Mix was prepared by adding 5.0 μL SYBR, 0.5 μL each of forward and reverse primers (500 nm), 1.5 μL of cDNA(5 ng/μL), and 2.5 μL of nuclease‐free water. RT‐PCR was performed in duplicate of 10‐μL reactions for each well. The reactions were run on the StepOnePlus time PCR system (Applied Biosystems), and the thermal cycling parameter was set to 95°C for 30 s followed by 40 cycles of 95°C for 15 s and then 60°C for 30 s. Relative gene expression changes were calculated using the ΔΔ Ct method given in StepOnePlus 3.0 software). For the identification of the suitable reference gene, various housekeeping genes were evaluated by using BestKeeper software that was based on the standard deviation of the crossing points [25]. Housekeeping genes such as *GAPDH, UBQ, RPS*, and β‐actin were used for the study, and finally, the *GAPDH* gene was chosen as a suitable housekeeping gene for further studies. The gene expression was normalized with relative to the expression of endogenous *GAPDH*. All the primers were designed using Primer 3 Plus 4.0 software [26]. The primer sequences and details are mentioned in Table 1. The experiment was replicated six times.

**TABLE 1:** The sequence of primers for qPCR.

Genes	Primer sequence (5′–3′)	GenBank Accession No.	Product size (bp)	Annealing temperature
GAPDH	F‐GGGTCATCATCTCTGCACCT R‐GGTCATAAGTCCCTCCACGA	NM_001190390.1	176	58.90 57.77

BAX	F‐ TCGGAGATGAATTGGACAGTA R‐CAGTTTGCTGGCAAAGTAGAA	XM_027978594.1	164	59.87 60.03

BCL2	F‐TGTGGAGAGCGTCAACCG R‐ACAAAGGCGTCCCAGCC	NM_001009226.2	113	58.90 58.25

CASP 3	F‐GATCTGGTACAGACGTGGATG R‐AACTGCTCCTTTTGCTATGGT	NM_001286089.1	150	57.99 57.77

ESR 1	F‐GGTTCCGTATGATGAATCT R‐ CAAGGTGTCTGTGATCTT	XM_027972563.1	197	58.90 57.77

ESR 2	F‐TGTCATCAGAGGCAGAGTTTG R‐CACACAAAGGAATCAAAGGGC	NM_001009737.1	130	60.13 60.06

PGR	F‐ATGGTTCTTGGAGGTCGAAAG R‐GTTTGCTGTTGTCATGTCCTG	XM_015100878.3	229	57.94 58.00

FSHR	F‐TCCTCTCCTACTTTGGGACAC R‐AAAGTCCAAGGCAGTTGATG	NM_001009289.1	196	58.10 57.80

CYP 11 A1	F‐AGACTTGGAGGGACCATGTAG R‐AATATTGGCCTTGACATCCTC	NM_001093789.1	152	57.30 58.34

CYP 17 A1	F‐CAGACAATAACAACACTGGC R‐GGTCAATGCTATCCTGGATCC	NM_001009483.1	215	57.66 57.36

CYP19 A1	F‐GCTTTTGGAAGTGCTGAACC R‐CTGGGACCTGGTATTGAGGA	NM_001123000.1	172	58.10 57.58

StAR	F‐ GGGCCCTGGGCATCCTCAAAGA R‐ TGACACTGGGGTTCCACTCGCCC	NM_001009243.1	194	58.36 57.30

3βHSD1	F‐TGCAAGTTCTCCAGAGTCAAC R‐GTCATCATAGCTTTGGTGTGG	NM_001135932.1	229	58.36 57.30

17βHSD1	F‐CTGGACAACCTTTCCCTTGA R‐GGCTGAAGGAAGCAAAACAG	NM_001267882.1	214	58.36 57.36

*Note: GAPDH*: glyceraldehyde‐3‐phosphate dehydrogenase, *ESR 1*: estrogen receptor 1 (alpha), *ESR2*: estrogen receptor 1 (beta), *PGR*: progesterone receptor, *CYP19 A1*: cytochrome P450 aromatase, *3β-HSD*: 3β‐hydroxysteroid dehydrogenase 1, *β17- HSD*: 17β‐hydroxysteroid dehydrogenase 1, *CYP 11 A1*: cytochrome P450 steroid 11‐α‐hydroxylase 1, *CYP 17 A1*: cytochrome P450 steroid 17‐α‐hydroxylase 1, *StAR*: steroidogenic acute regulatory protein, *BAX*: *BCL2*‐associated X protein, *BCL2*: B‐cell lymphoma 2 protein, *CASP3*: *Caspase-3*.

Abbreviation: *FSHR*, follicle‐stimulating hormone receptor.

### 2.15. Statistical Analysis

The experimental data from in vitro studies and genomic analyses were evaluated using the general linear model with repeated measures analysis of variance (ANOVA) in SPSS version 16.0. The results were reported as the mean ± standard error (SE) from 10 different replicates. Data were subjected to one‐way ANOVA after confirming homogeneity of variances using Levene’s test. Post hoc comparisons among means were performed using Tukey’s HSD test at a significance level of *p* < 0.05.

## 3. Results

### 3.1. Apoptosis Assay Through Nuclear Staining

Various nuclear morphological attributes, including condensed nuclei, membrane blebbing, and apoptotic bodies, were observed in BPA‐treated GCs. Following Hoechst staining and treatment with different BPA concentrations for 72 h, GCs displayed distinct apoptotic features such as cell shrinkage, nuclear condensation, and fragmentation (Figures 1(c), 1(d), 1(e) and 1(f)). In contrast, control cells and lower concentration of BPA (1 μm)‐treated GCs maintained a regular morphology with intact nuclear architecture (Figures 1(a) and 1(b)), respectively. These apoptotic changes are attributed to the activation of caspase cascades, which cleave specific substrates involved in DNA repair mechanisms.

FIGURE 1Fluorescent images of Hoechst staining showing BPA‐induced cell death. Treatment of BPA with (a) 0 μm (control), (b) 1 μm, (c) 10 μm, (d) 25 μm, (e) 50 μm, and (f) 100 μm on GCs cultured for 72 h. The arrows indicate the formation of apoptotic bodies. Scale bar = 50 μm.

**Figure:**
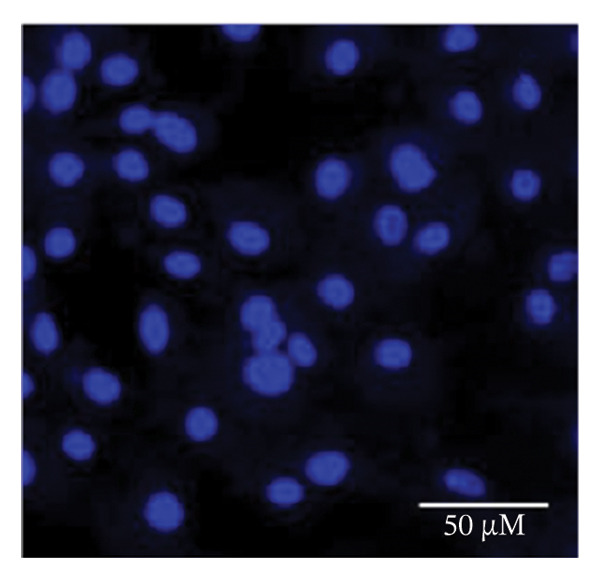
(a)

**Figure:**
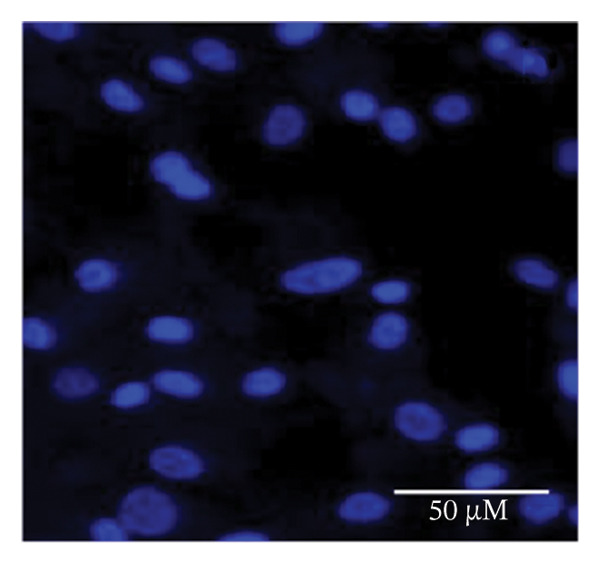
(b)

**Figure:**
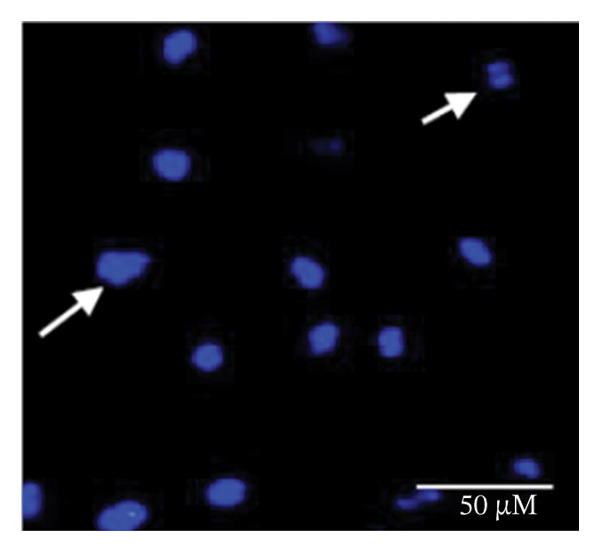
(c)

**Figure:**
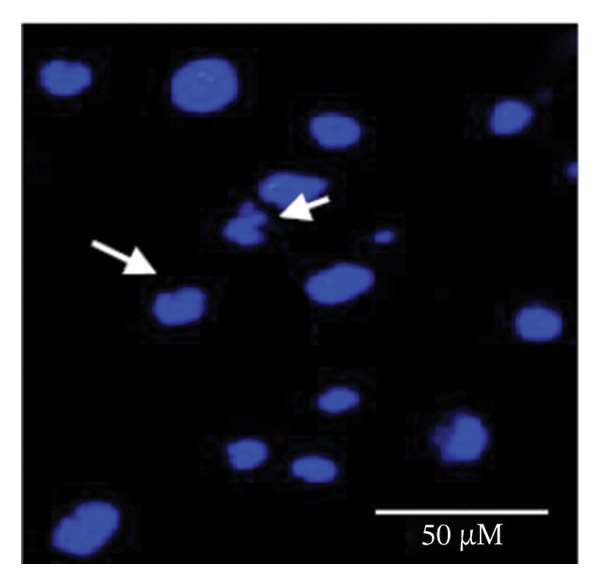
(d)

**Figure:**
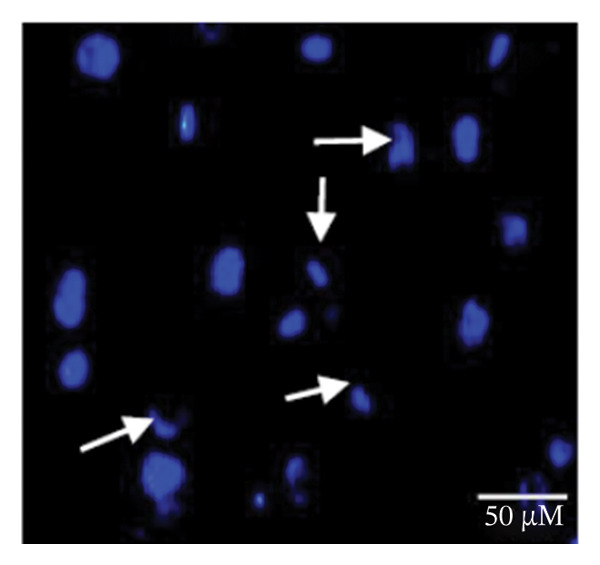
(e)

**Figure:**
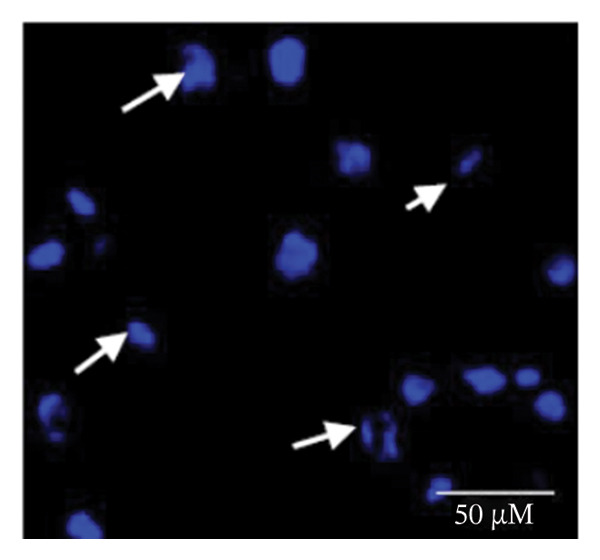
(f)

### 3.2. Effect of Various Levels of BPA on GC Growth Parameters of Sheep

The metabolic activity (AU/10^5^) of GCs in a 3‐day culture after exposure to BPA showed a nonsignificant change up to a concentration of 25 μm compared to those observed in lower concentration groups (Table 2). However, there was a significant reduction in the metabolic activity of GCs at a 50‐μm dose of BPA and did not show any significant change in the metabolic activity despite increasing the BPA concentration to 100 μm. The viability rate (%) of GCs in a 3‐day culture revealed a significant reduction at a 10‐μm dose of BPA, and no further improvement was noted despite increasing the dose of BPA to 100 μm compared to lower concentrations. The rate of monolayer formation in the GCs after exposure to the BPA in a 5‐day culture revealed a significant reduction at the 1‐μm dose of BPA, and no further improvement was noted as the dose of BPA was increased to 100 μm. The cell number increment of GCs exposed to BPA in a 2‐day culture revealed a significant reduction at a 1‐μm dose and did not show any significant change up to a concentration of 50 μm compared to lower treatment groups. However, a significant reduction was again noted in the cell number increment of GCs at 100 μm of BPA exposure (Table 2).

**TABLE 2:** Effect of various levels of bisphenol A (BPA) on ovine GC growth, viability, monolayer formation, cell number increment, and hormone production *in vitro*.

Treatment BPA (μm)	Metabolic activity of GC absorbance units (AU)/10^5^ cells 3‐day culture	Viability of GCs (%) 3‐day culture	Rate of monolayer formation 5‐day culture	Cell no. increment (× 10^5^) 2‐day culture	Estrogen (pg/mL) 3‐day culture	Progesterone (ng/mL) 3‐day culture
Control (0 μm)	0.68 ± 0.08^b^	92.89 ± 0.26^c^	1.94 ± 0.09^b^	1.53 ± 0.02^c^	54.99 ± 1.71^b^	9.78 ± 0.43^b^
1	0.57 ± 0.0^ab^	75.22 ± 5.53^bc^	1.60 ± 0.06^a^	1.38 ± 0.02^b^	64.27 ± 0.98^c^	12.00 ± 0.53^c^
10	0.53 ± 0.03^ab^	70.78 ± 5.52^ab^	1.62 ± 0.02^a^	1.40 ± 0.02^b^	71.46 ± 1.28^d^	12.71 ± 0.30^c^
25	0.50 ± 0.03^ab^	65.22 ± 5.51^ab^	1.66 ± 0.09^ab^	1.37 ± 0.02^b^	41.01 ± 1.41^a^	9.56 ± 0.26^b^
50	0.47 ± 0.01^a^	64.56 ± 2.82^ab^	1.58 ± 0.07^a^	1.35 ± 0.02^ab^	43.94 ± 1.58^a^	7.37 ± 0.30^a^
100	0.42 ± 0.04^a^	54.67 ± 6.77^a^	1.59 ± 0.04^a^	1.28 ± 0.01^a^	40.04 ± 1.60^a^	7.88 ± 0.34^a^

*Note:* Values are mean ± S.E.M based on 15 replicates per treatment and consist of 1 × 10^5^ GCs per culture drop.

^abcd^Superscripts within the same column differ significantly (*p* < 0.05).

### 3.3. Effect of Various Levels of BPA on Hormone Concentration in the Spent Media of GC Cultures

The concentration of 17β‐estradiol in the spent media of GCs cultured with BPA at various concentrations revealed a significant increment at the 1‐μm level of BPA with a further increment up to 10 μm compared to those observed in the lower concentration groups. However, a significant reduction in the estradiol concentration of the spent media of the GCs cultured with BPA was noted at a 25‐μm level and did not show any significant change further despite increasing the dose of BPA to 100 μm (Table 2). The progesterone concentration in the spent media of GC cultured with BPA revealed a significant increment at the 1‐μm level compared to those of the control group. However, a significant reduction in the progesterone concentration of spent media from the GCs cultured with BPA was noted at a 25‐μM level, with a further reduction at a 50‐μM concentration compared to the lower treatment groups (Table 2).

### 3.4. Effect of Various Levels of BPA on Cytotoxic Assays of Cultured GCs in Sheep

The estimation of LDH assay (% cytotoxicity) in the GCs cultured with BPA revealed significantly higher values from 50 μm onwards compared to lower treatment groups (Table 3). The ROS (μm) in the GCs revealed significantly higher values at the 10‐μm level of BPA and did not show any significant change despite increasing the level of BPA up to 100 μm compared to lower concentrations (Table 3). The values obtained with the MDA (μm) assay in the GCs exposed to BPA were significantly higher than those exposed to a 25‐μm level of BPA. However, a significant reduction was noted in the MDA values at the 50‐μm level of BPA, and no significant difference was noted further despite increasing the level of BPA to 100 μm (Table 3). The activity of the CUPRAC assay (μm) was nonsignificant at all the experimental concentrations (Table 3). The activity of the GST assay (μmole/mL/min) in the GCs exposed to BPA was significantly lower at the 50‐μm level compared to the lower treatment groups and did not show any significant difference in the GST activity, although the level of BPA increased further to 100 μm (Table 3).

**TABLE 3:** Effect of different levels of bisphenol A (BPA) on ovine GC cytotoxicity (LDH), oxidative stress (ROS), lipid peroxidation (MDA), total antioxidant activity (CUPRAC), and detoxification activity (GST).

Treatment BPA (μm)	LDH (% cytotoxicity) 3‐day culture	ROS H_2_O_2_ (μm) 3‐day culture	MDA (μm) 3‐day culture	CUPRAC activity (μm) 3‐day culture	GST activity (μmole/mL/min) 3‐day culture
Control (0 μm)	8.2 ± 1.55^a^	20.19 ± 1.95^a^	1.91 ± 0.23^a^	42.52 ± 5.43^a^	76.00 ± 3.84^b^
1	6.7 ± 1.12^a^	26.36 ± 1.54^ab^	2.17 ± 0.22^a^	44.10 ± 3.78^a^	74.67 ± 4.34^b^
10	9.5 ± 1.32^a^	27.55 ± 0.83^b^	2.32 ± 0.05^a^	42.82 ± 5.81^a^	76.67 ± 5.22^b^
25	9.8 ± 1.38^a^	29.16 ± 2.04^b^	3.51 ± 0.63^b^	42.20 ± 5.50^a^	71.50 ± 6.35^b^
50	17.6 ± 1.03^b^	31.69 ± 0.84^b^	2.48 ± 0.22^a^	47.52 ± 7.07^a^	55.83 ± 2.52^a^
100	18.5 ± 1.90^b^	31.81 ± 1.55^b^	2.62 ± 0.34^a^	46.84 ± 7.16^a^	60.83 ± 4.92^b^

*Note:* Values are mean ± S.E.M based on 15 replicates per treatment and consist of 1 × 10^5^ GCs per culture drop.

^ab^Superscripts within the same column differ significantly (*p* < 0.05).

### 3.5. Expression Profiles of Steroidogenesis Genes in the GCs Exposed to BPA at Various Concentrations

**FIGURE 2:**
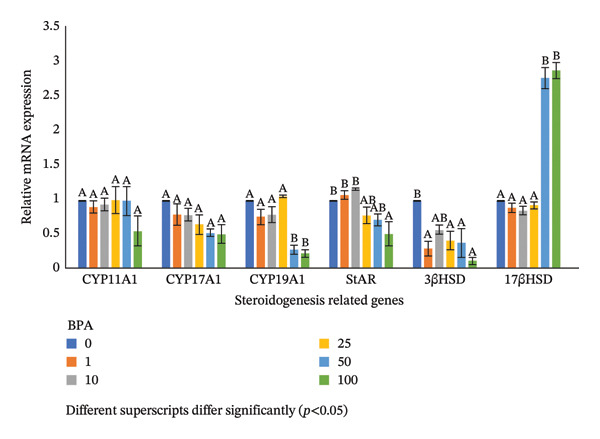
Expression profiles of steroidogenesis‐related genes in ovine GCs cultured in the presence of different levels of BPA.

The expression patterns of C*YP11A1* and *CYP17A1* in the cultured GCs exposed to BPA revealed a nonsignificant difference in all experimental levels compared to those observed in the control group (Figure 2). The C*YP19A1* gene expression was nonsignificant up to 25 μm of BPA. However, a significant downregulation was noted in the C*YP19A1* expression as the exposure dose of BPA increased to 50 μm and did not show any further significant change despite increasing the dose of BPA to 100 μm (Figure 2).

### 3.6. Expression Profiles of Steroidogenic Protein Genes in the GCs Exposed to BPA at Various Concentrations

The expression pattern of the StAR gene in the cultured GCs exposed to BPA was nonsignificant up to 50 μm compared to lower concentration groups. However, a downregulation in the *StAR* expression was noted as the dose of BPA increased to 100 μm (Figure 2). The gene expression of *3βHSD1* in the GCs exposed to BPA was significantly downregulated at a concentration of 1 μm compared to that observed in the control and did not show any significant change further despite increasing the level of BPA to 100 μm (Figure 2). The gene expression of *17βHSD* in the GCs exposed to BPA was nonsignificant in the GCs up to 25 μm exposure to BPA. However, there was a significant upregulation in the *17βHSD* expression exposed to 50 μm compared to lower concentrations and did not show any significant change despite increasing the level of BPA to 100 μm (Figure 2).

### 3.7. Expression Profiles of Hormone Receptor Genes in the GCs Exposed to BPA at Various Concentrations

**FIGURE 3:**
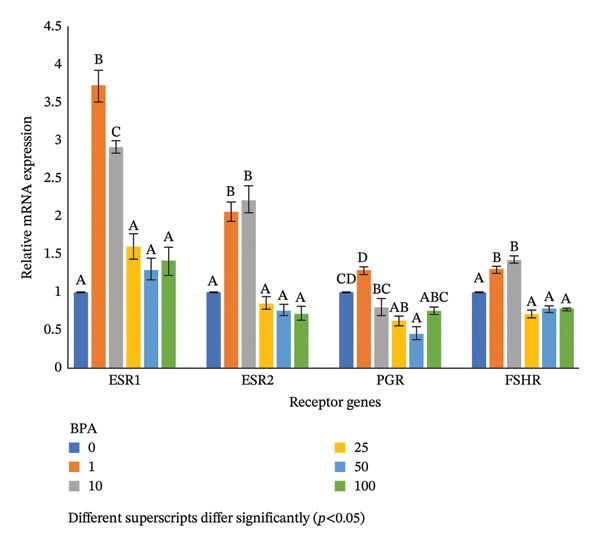
Expression profiles of steroidogenesis‐related receptor genes in ovine GCs cultured in the presence of different levels of BPA. Different superscripts differ significantly (*p* < 0.05).

Expression profiles of the *ESR1* gene in the GCs cultured with BPA were significantly upregulated at a 1‐μm level compared to the control. As the exposure dose of BPA increased further, there was a downregulation in the *ESR1* expression from 10 μm onwards up to 25 μm, which did not show any significant change despite increasing the level of BPA up to 100 μm compared to the lower concentrations (Figure 3). The *ESR2* gene expression was upregulated in the GCs exposed to BPA at 1 μm compared to the control group. However, a significant downregulation was noted at the 25‐μm level of BPA and did not show any significant change despite increasing the level of BPA up to the 100‐μm concentration compared to the lower concentrations (Figure 3). The *PGR* gene expression was downregulated in the GCs exposed to BPA at a 25‐μm concentration compared to lower concentrations and did not show any significant change up to 100 μm despite increasing the level of BPA (Figure 3). The *FSHR* gene expression was upregulated in the GCs exposed to BPA at 1 μm compared to the control group. However, a significant downregulation was noted at the 25‐μm dose of BPA and did not show any significant change despite increasing the level of BPA up to the 100‐μm concentration compared to the lower concentrations (Figure 3).

### 3.8. Expression Profiles of Apoptotic‐Related Genes in the GCs Exposed to BPA at Various Concentrations

**FIGURE 4:**
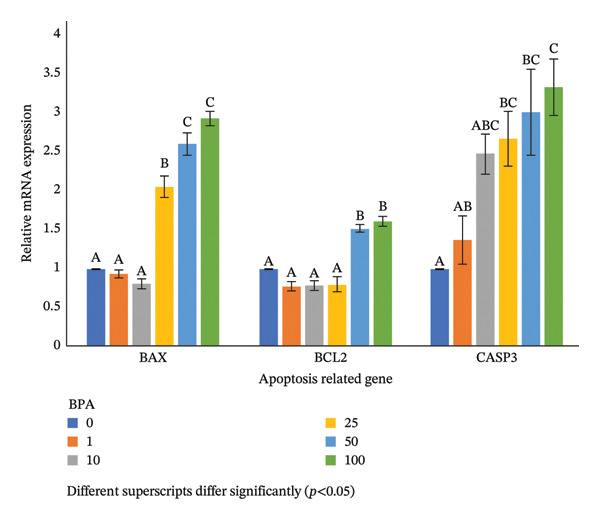
Expression profiles of apoptosis‐related genes in ovine GCs cultured in the presence of different levels of BPA. Different superscripts differ significantly (*p* < 0.05).

The expression profile of *BAX* in the GCs exposed to BPA revealed a significant upregulation from 25 μm onwards and up to the 50‐μm dose compared to lower concentrations and did not show any significant change in the *BAX* expression despite increasing the level of BPA to 100 μm (Figure 4). The expression profile of *BCL2* in the GCs exposed to BPA revealed a significant upregulation at the 50‐μm level compared to lower concentration groups and did not show any significant change in the *BCL2* expression despite increasing the level of BPA to 100 μm (Figure 4). The expression profile of *CASP3* in the GCs exposed to BPA revealed a significant upregulation at a 25‐μm level compared to lower concentrations and did not show any significant change in the *CASP3* expression despite increasing the level of BPA to 100 μm (Figure 4).

## 4. Discussion

The present investigation revealed that ovine ovarian GCs endured negative consequences when exposed to BPA in vitro. In the present study, we used MTT (mitochondrial disintegration), LDH (plasma membrane damage), MDA (lipid peroxidation), and ROS (oxidative damage) assays as toxicological endpoints to measure the cytotoxicity and oxidative stress of GCs in addition to the growth and/or proliferation rate of BPA on GCs. To further corroborate the findings, the expression of genes involved in steroidogenesis and apoptosis was assessed. In agreement with previous studies, our findings corroborated that both BPA and BPS exerted deleterious effects on ovine GCs through mechanisms involving oxidative stress, mitochondrial dysfunction, and disrupted steroidogenic signaling. It was demonstrated that bisphenol exposure induced a dose‐dependent reduction in progesterone and estradiol synthesis, accompanied by the downregulation of *StAR* and *CYP19A1* transcripts and increased expression of apoptotic markers *BAX* and *CASP3* [11, 17]. Our findings showed similar elevation in the *BAX* and *CASP3* expression consistent with enhanced apoptosis, but in contrast, a rise in progesterone and estradiol secretion, indicating a divergent, possibly compensatory, steroidogenic response under bisphenol‐induced stress. BPS, often marketed as a safer substitute, induced comparable or stronger perturbations in the redox balance and estrogen receptor expression [18]. The placental transfer and fetal accumulation also highlighted the potential for *in vivo* exposure and cumulative reproductive toxicity in small ruminants [10]. Therefore, the consistent disruption of endocrine, oxidative, and apoptotic pathways across studies supported the notion that bisphenols collectively compromise ovarian cellular integrity and reproductive efficiency in domestic animal species.

The effect of BPA on GCs was in a single direction and decreased (negative monotonic) with increasing dose. Although the response was accelerated at a higher dose, it was steeper. The reduction in the GC proliferation and viability in the ovine (50, 100, and 200 μm) [11], bovine (5 and 50 μg/mL) [27], and mouse (100 ppm) [28] GCs were also reported after exposure to BPA. The molecular mechanism behind inhibiting GC proliferation by BPA was increased ROS production [27], increased proapoptotic Bax protein level [28], and/or increased cell apoptosis by reducing mitochondrial membrane potential [29].

Therefore, with the assumption of the cytotoxic effects of BPA on GCs, we estimated the various cytotoxic assays in the GCs exposed to BPA in an in vitro culture system in the present study. It is well known that the extent of the cytotoxicity of BPA on the cell membrane can be measured by various cytotoxic assays. Among them, LDH is a stable enzyme that leaks from the cell in relatively high amounts upon cell plasma membrane damage [30]. The LDH was higher in the GCs exposed to BPA in the present study, which has a direct correlation with GC membrane damage. The possible reason behind higher LDH in the present experiment might be due to the alterations (*CCND2:* downregulation; *CDK4* and *CCNE1*: upregulation) [31, 32] of cell cycle regulators at the G1‐S phase and/or creating an imbalance in the ROS concentration. In the present study, we also found a higher level of ROS and MDA concentrations in the GCs after BPA exposure. This demonstrated an oxidative damage and lipid peroxidation respectively to the cell membrane after exposure to BPA. Induction of ROS in the GCs upon exposure to BPA also reported in Ref. [28] (murine) and Ref. [27] (bovine), and the authors suggested that inducing cell cycle arrest (G2 to M), apoptosis, and endocrine disruption could be an attributable reason. The result of the present study is comparable in this regard. However, several antioxidant systems regulate ROS levels in the cell and defend them from oxidative damage. Among them, GST is well known. Hence, we also estimated the activity of GST in the GCs exposed to BPA in the present experiment. The lower activity of GST in our experiment demonstrated cellular stress induced by BPA, which in turn could hamper antioxidant enzyme formation. The altered expression of antioxidant enzymes (GST and GSH) by BPA exposure (1 μm) in the GCs was also reported in *in vitro* studies [33]. To confirm this, further total antioxidant activity (CUPRAC) was further studied. However, we did not find any considerable change in the total antioxidant activity with different concentrations of BPA in the present experiment. In consequence, higher apoptosis can be induced in the GCs compared to the normal process, which was evidenced in our study. Similar results of ovarian cell apoptosis were reported in the GCs exposed to BPA (200 μm) in the ovine after 48 h of culture [11]. In the present study, it is posited that the imbalance between ROS production and cellular coping mechanisms led to the overwhelming of antioxidant defense systems in GCs upon exposure to BPA. This induced oxidative stress hampered cell survival and ultimately decreased the cell viability.

To further investigate the GC growth parameters after BPA exposure, various hormone concentrations were measured in the spent media of GC cultures. The data revealed an inverted *U*‐shaped nonmonotonic dose–response (NMD) curve for both estradiol and progesterone concentrations. The estradiol concentration in the spent media initially increased at lower BPA concentrations (1–10 μm) but decreased with higher BPA exposure (25 μm). This indicates that BPA can act as both an estrogen agonist (at lower concentrations) and an antagonist (at higher concentrations). This biphasic effect occurs because BPA binds to endogenous estrogen receptors (ERα and ERβ) with high affinity, altering their function. Specifically, BPA inhibits the ERβ ligand‐binding domain (LBD) from assuming the correct conformation while causing a similar displacement of α‐helices in the ERα LBD as estradiol (E2) does [34]. BPA’s estrogenic effects in GC cultures were reported in Ref. [35] in humans (10 μm for 24 h), and its proliferative effect was reported in Ref. [36] in human HBL cell line (1.0 × 10^−6^ mol/L for 6 days). Conversely, its antiestrogenic effects were observed in ovine (50, 100, and 200 μm for 48 h) [11] and in murine (10^−6^ to 10^−4^ M for 48 h) [37].

Similarly, the progesterone concentration in the GC cultures exhibited an inverted *U*‐shaped NMD response. Progesterone levels initially increased at lower BPA doses but decreased at higher doses. This biphasic response is likely due to the downregulation of steroidogenesis enzymes at higher BPA concentrations [38]. Higher progesterone levels in GCs exposed to BPA were reported in porcine (10^−8^ to 10^−5^ M) in Ref. [39], while lower levels were noted in porcine (100 μm) in Ref. [15]. Biphasic responses similar to those observed in our study were reported in porcine (0.1–10 μm) [40] and in murine (10^−5^ and 10^−4^ M) [37] in ovarian GCs after BPA exposure. The reason behind this inhibition can be attributed to its affinity for endogenous receptors (ERs) and the expression of a phenol group resembling estradiol [41]. These results collectively demonstrate that BPA’s effects on GC growth parameters are dose‐dependent, further establishing its role as an endocrine‐disrupting chemical. These findings support our results, showing that BPA can reduce GC growth parameters.

Furthermore, to investigate the biphasic effects of BPA on GCs, expression profiles of mRNA of genes responsible for the steroidogenesis and steroidogenic proteins were studied in the present study. The downregulation of C*YP19A1* (50 μm) and *StAR* (100 μm) in the GCs at higher doses of BPA exposure can be due to various reasons such as suppression of protein (FSHR/Gαs)/adenylyl cyclase signaling pathway [42] and/or impedance of certain transcription factors (SF‐1: steroidogenic factor‐1, GATA‐4 and PPAR‐γ: peroxisome proliferator‐activated receptor gamma) in the process of steroidogenesis exposed to BPA [43]. A similar report was published in Ref. [44], where a reduction in the steroid hormone synthesis is observed in the mouse antral follicles exposed to BPA (10 and 100 μg/mL) due to the lower expression of *StAR* and *CYP11A1* through ERK (extracellular signal‐regulated kinases) and/or DAX‐1 (nuclear receptor protein) signaling pathways and second messenger signals. However, a downregulation of *CYP11A1* and an upregulation of *StAR* were reported in the ovarian GCs exposed to 10^−4^ M concentration of BPA. In the present study, however, a nonsignificant change was noted in the *CYP11A1* and *CYP17A1* expression [37]. The variation obtained in the present study was probably due to the species and dose differences [37]. The upregulation of the *17βHSD* expression (50 μm) in the present study was possibly due to binding of its transcription factors more precisely by BPA, which assisted in the uncontrolled expression of the genes. Similarly, a downregulation was observed in the *3βHSD* expression at the higher level of exposure (100 μm) of BPA in the mRNA of GCs of murine [11].

The effects of BPA on hormone receptors have also been studied in the present study as BPA is known to be an estrogenic compound and can bind to and act through *ESR1* and *ESR2* receptors [45]. The upregulation of both the receptors (*ESR1* and *ESR2*) in the GCs of mRNA was noted in the present study exposed to even lower level (1 μm), suggesting a stimulatory effect of BPA. However, a downregulation was also noted in both the receptors as the dose of exposure increases (25 μm). This is in correlation with the concentration of estradiol obtained in the present study, where a biphasic effect was observed due to BPA exposure by binding to the endogenous estrogen receptors (*ERα* and *ERβ*). The probable reason behind the downregulation of the estrogen receptor gene (*ESR1* and *ESR2*) expression in the present study after exposure to BPA could be a reduction in the MAPK3/1 phosphorylation in the membrane [17]. Likewise, progesterone receptor is held responsible in the progesterone hormone synthesis, which is one of the main hormones that regulated ovulation and other reproductive processes. A significant improvement was observed in the *PGR* gene mRNA expression by exposing the GCs at 10 μm concentrations in the ovine [17]. The expression pattern of the *FSHR* gene also demonstrates a biphasic effect in this study. Our findings indicate that BPA may exert an estrogenic influence on the *FSHR* expression, suggesting that BPA can impact *FSHR* signaling at various levels. BPA may directly target receptors expressed on the ovarian cell surface, acting as allosteric modulators and binding provocatively [46]. This interaction may up‐ or downregulate the receptor expression, indicating a disruption in the FSH signal that alters communication with *FSHR* membrane partners. Consequently, this disturbance affects the physical interaction and communication between intracellular signaling pathways [46].

Nevertheless, measuring apoptosis‐related genes in GCs exposed to BPA provides valuable insights into the extent of apoptosis. In the present study, GCs’ viability decreased and apoptosis increased at higher BPA doses (> 25 μm). This phenomenon was likely caused by elevated levels of apoptotic proteins, such as *BAX* and *CASP3*, and altered levels of the antiapoptotic protein *BCL-2*. The higher Bax gene expression in cultured GCs exposed to higher BPA doses reflected this trend. The higher *CASP3* expression indicated irreversible cell death at higher BPA concentrations, likely due to the activation of the proteolytic cascade. These findings suggest that BPA at concentrations greater than 25 μm can act as an external trigger to induce apoptosis. In the present study, a linear increase in the *BAX/BCL2* ratio in GCs exposed to higher BPA doses further demonstrated increased apoptosis. Supporting these findings, it was demonstrated that the exposure of GCs to 100‐μm BPA increased the BAX/BCL2 ratio in murine, leading to DNA damage [27]. Furthermore, BPA can disrupt the mitochondrial function, thereby triggering the intrinsic pathway of apoptosis in bovine GCs [47]. Overall, these findings underscore the toxic effects of BPA on ovarian cell viability and function, highlighting the need for further investigation into safer alternatives to BPA in consumer products.

## 5. Conclusion

**FIGURE 5:**
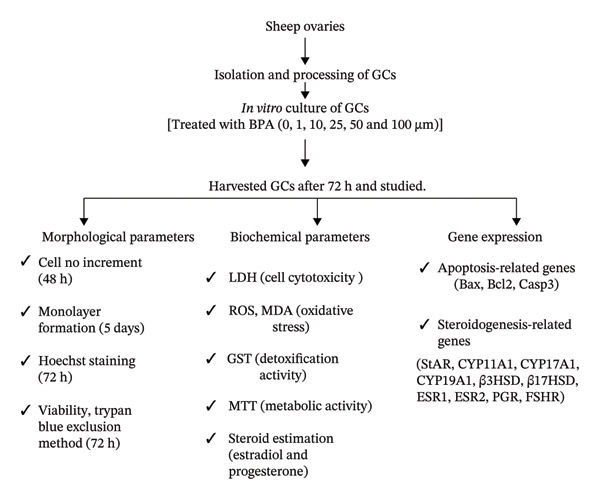
Schematic diagram of experimental design for workflow. Different superscripts differ significantly (*p* < 0.05).

BPA inhibits GC growth and proliferation at doses of 1–50 μm, with stronger effects at higher concentrations. It induces oxidative stress, increases ROS, and reduces antioxidant activity. Hormonal analysis shows a biphasic response, acting as both an estrogen and progesterone agonist and antagonist depending on the dose. These findings highlight BPA’s disruptive impact on ovarian function and the need for safer alternatives. Future studies should integrate organelle‐specific assessments, particularly mitochondrial and endoplasmic reticulum dynamics, along with detailed proteomic profiling to unravel the mechanistic protein‐level alterations underlying BPA‐induced GC dysfunctions (see Figure 5).

## Author Contributions

Poonam Kumari Singh and Bogapathi Sampath Kumar contributed to the execution of the study and manuscript drafting, and Sumanta Nandi contributed to the design and critically revised the manuscript. Paluru Subramniyam Parameswara Gupta contributed to the analysis of data, and Sukanta Mondal contributed to the PCR studies.

## Funding

We are grateful to the University Grants Commission (UGC), New Delhi, India [Ref. No. 21/06/2015 (i) EU‐V], for funding and institutional funding.

## Conflicts of Interest

The authors declare no conflicts of interest.

## Data Availability

The data that support the findings of this study are available from the first author upon reasonable request.
